# Evaluation of Algebraic Iterative Image Reconstruction Methods for Tetrahedron Beam Computed Tomography Systems

**DOI:** 10.1155/2013/609704

**Published:** 2013-05-27

**Authors:** Joshua Kim, Huaiqun Guan, David Gersten, Tiezhi Zhang

**Affiliations:** ^1^TetraImaging, 4591 Bentley Drive, Troy, MI 48098, USA; ^2^Department of Physics, Oakland University, 2200 N. Squirrel Road, Rochester, MI 48309, USA; ^3^21st Century Oncology Inc., 4274 W. Main Street, Dothan, AL 36305, USA; ^4^Department of Radiation Oncology, William Beaumont Hospital, 3601 W. Thirteen Mile Road, Royal Oak, MI 48073, USA

## Abstract

Tetrahedron beam computed tomography (TBCT) performs volumetric imaging using a stack of fan beams generated by a multiple pixel X-ray source. While the TBCT system was designed to overcome the scatter and detector issues faced by cone beam computed tomography (CBCT), it still suffers the same large cone angle artifacts as CBCT due to the use of approximate reconstruction algorithms. It has been shown that iterative reconstruction algorithms are better able to model irregular system geometries and that algebraic iterative algorithms in particular have been able to reduce cone artifacts appearing at large cone angles. In this paper, the SART algorithm is modified for the use with the different TBCT geometries and is tested using both simulated projection data and data acquired using the TBCT benchtop system. The modified SART reconstruction algorithms were able to mitigate the effects of using data generated at large cone angles and were also able to reconstruct CT images without the introduction of artifacts due to either the longitudinal or transverse truncation in the data sets. Algebraic iterative reconstruction can be especially useful for dual-source dual-detector TBCT, wherein the cone angle is the largest in the center of the field of view.

## 1. Introduction

Image-guided radiation therapy (IGRT) is essential to ensure proper dose delivery to the target while sparing the surrounding tissue [1, 2]. Cone beam CT (CBCT) is a popular online imaging modality used for LINAC-based IGRT [3, 4]. Although CBCT is convenient to use, the performance of CBCT systems is less than ideal. The image quality for the CBCT is significantly degraded due to excessive scattered photons [5–8] as well as suboptimal performance of the flat panel detector [9]. These issues limit the use of CBCT for certain advanced radiation therapy techniques such as online adaptive radiotherapy [8, 10]. It is also well known that at large cone angles, there are artifacts caused by using approximate reconstruction methods that appear in CBCT reconstructions [11], but this issue has largely been ignored in IGRT because the scatter and detector issues are the dominant factors in the degradation of CBCT image quality.

Tetrahedron beam computed tomography (TBCT) is a novel volumetric CT modality that overcomes the scatter and detector problems of CBCT [12, 13]. A TBCT system is composed of a minimum of one linear source array with one linear detector array positioned opposite and orthogonal to it. In TBCT, scattered photons are largely rejected due to the fan-beam geometry of the system. A TBCT system also uses the same high performance detectors that are used for helical CT scanners. Therefore, TBCT should be equivalent to diagnostic helical CT with regard to scatter rejection and detector performance. However, similar to CBCT, the data sufficiency condition [14, 15] is not satisfied with a single axial TBCT scan. TBCT still suffers from the same large cone angle artifacts that are present in CBCT images reconstructed using the conventional Feldkamp-Davis-Kress (FDK)-type approximate filtered backprojection (FBP) algorithm [16]. More importantly, in a TBCT system that is composed of two source arrays and two detector arrays, the cone reconstruction artifact is most significant in the center of the field of view (FOV). Therefore, reducing cone artifacts is more important for this arrangement.

Owing to the rapid improvement in computational power, it has become practical to use iterative reconstruction methods in the clinic. Iterative imaging reconstruction methods have been proven to be capable of reducing imaging dose [17, 18], increasing image resolution [19, 20], and reducing artifacts [21, 22]. Most CT vendors provide different iterative image reconstruction solutions for their diagnostic CT scanners. The algebraic reconstruction technique (ART) [23] and the simultaneous ART (SART) [24] algebraic iterative methods, in particular, have been shown to reconstruct cone beam data with minimal artifacts at large cone angles [21].

In order to further improve TBCT image quality and reduce reconstruction artifacts at larger cone angles, we implemented iterative algebraic reconstruction methods for different TBCT geometries in this study. We evaluated the performance of these algorithms using various numerical phantoms as well as digitally-projected patient images. The patient reconstruction results were then compared to the reconstructed images produced using a fan-beam reconstruction method that was considered to be the ground truth for this study.

## 2. Material and Methods

### 2.1. TBCT Geometries

**Figure 1:**
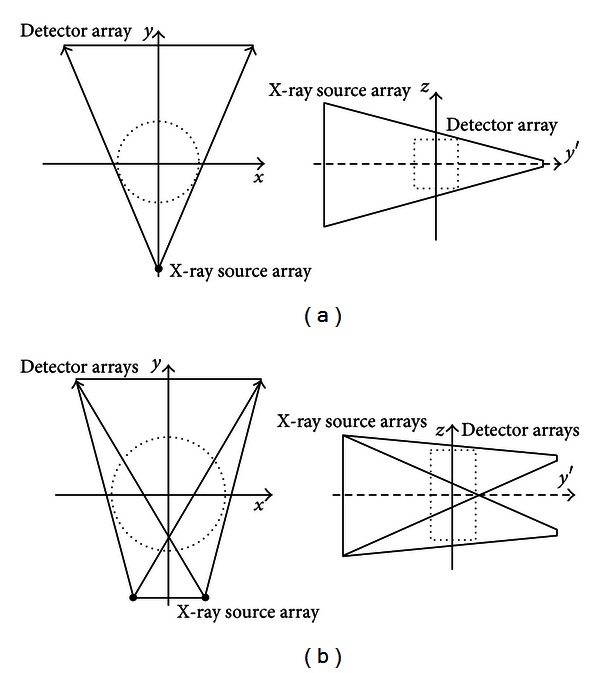
Single-source single-detector TBCT geometry (a). Dual-source dual-detector TBCT geometry (b).

The TBCT system geometry is flexible enough to incorporate multiple source and detector arrays if the need arises.  Figure 1 shows a comparison of the geometries for the [single-source single-detector] TBCT system and for the dual-source dual-detector TBCT system. With the dual-source dual-detector geometry, the length of the detector and source arrays can be reduced while still being able to achieve the same FOV. However, for a TBCT system that uses two detector arrays, the approximate reconstruction artifacts would be most prominent in the central transverse plane of the image instead of at the top and bottom of the image. Therefore, reducing the cone artifact is especially important for the [dual-source dual-detector] TBCT system.

### 2.2. Algebraic Reconstruction Algorithms

Detector projection measurements during a CT scan are represented by the linear system equation

(1) $$\begin{array}{c} p=Ax, \end{array}$$

where **x** = (*x*_1_, *x*_2_,…, *x*_*N*_) represents the image to be reconstructed and **p** = (*p*_1_, *p*_2_,…, *p*_*M*_) represents the measured projection data. *M* and *N* are the total number of line integral measurements and total number of image voxels, respectively. The total number of measurements, *M*, is the product of the number of detectors and the number of projections per detector. The system matrix **A** ∈ ℝ^*M*×*N*^ has matrix elements *a*_*ij*_ ≥ 0 that map the image voxel *i* onto the projection measurement *j*. In iterative reconstruction, the image voxel values are treated as unknowns in the system of equations given by (1). For a 3D CT scan, the dimensions of the system matrix **A** are enormous. Both *M* and *N* could be in the order of hundreds of millions.

To calculate the elements of the system matrix, we implemented the distance-driven method introduced by De Man and Basu [25]. For this method, the boundaries of the detectors and voxels are mapped onto a common plane. The lengths of overlap of the detector and voxel boundaries along each of the axes of the plane are then calculated. These two values are then multiplied together to determine the value of the system matrix elements.

This system of equations cannot be solved directly due to the ill-posedness of the problem, the noise in the data, and the immense size of the system matrix, but it can be solved iteratively using an algebraic approach. Iterative methods begin with an initial guess of the image voxel values, which is then forward projected using the system matrix to produce an estimate of the projection data. The differences between the estimated and measured projection data are calculated and used to determine correction terms which are then back projected onto the image. This process is iteratively repeated until some convergence criteria have been satisfied or a preset number of iterations has completed. For this study, we have chosen to implement the well-known SART algorithm [24] which has been shown to converge to the weighted least squares solution from any initial guess [26]. It has also been demonstrated in previous studies that the convergence of algebraic methods could be improved by varying the order in which projections are processed [27], and so we implement the SART both with and without the use of the multilevel access ordering scheme (MAS) developed by Guan and Gordon [27].

#### 2.2.1. Simultaneous Algebraic Reconstruction Technique

The forward projection of each measurement is calculated using ${\hat{p}}_{j}=\sum_{i}a_{ij}x_{i}$ and then compared to the measured projection value *p*_*j*_. The difference between these values is then weighted and backprojected over the image. For the SART algorithm, all projection measurements collected at a single projection image are used to simultaneously update each image voxel value. The update term is given by

(2) $$\begin{array}{c} x_{i}^{n+1}=x_{i}^{n}+\lambda \frac{\sum_{j=A}^{B}a_{ij}\left(\left(p_{j}-{\hat{p}}_{j}\right)/\sum_{i=1}^{N}a_{ij}\right)}{\sum_{j=A}^{B}a_{ij}}, \end{array}$$

where *n* is the update step, *i* is the image voxel index, *j* is the projection data index, *λ* is the relaxation parameter, and *A* and *B* are the indices of the first and last projection data elements used for the *n*th update step. These values are defined by *A* = (*n* − 1)*K* and *B* = *nK* where *K* is the number of projection data elements that make up a single projection image. One iteration is completed after all projection images have been used to update the image. The image **x** converges to a stable solution after a few iterations. The relaxation parameter value that was chosen for this study was 0.08, which was selected by trial and error and falls within the range suggested in the literature [28, 29].

#### 2.2.2. Multilevel Projection Ordering Scheme

The MAS ordering system was developed for algebraic reconstruction algorithms in order to minimize the correlation between sequential projection images that are used to update the image [27]. This leads to an improvement in the convergence speed of the algebraic methods. This method has been evaluated and compared against alternate ordering systems and has been shown to provide the greatest benefit in improving the efficiency of the reconstruction algorithms [30]. For a system with *V* projection views ordered sequentially as 0, 1, …, *V* − 1, this system determines a number of levels according to *L* = log⁡_2_ *V*. If *V* is not a power of two, then one is added to the number of levels. The levels are ordered so that any two sequential views are chosen for maximum orthogonality between them. The order of the indices in the first level is set as 0 (0°) followed by *V*/2 (90°). The second level again has two elements and the indices are set as *V*/4 (45°) followed by 3*V*/4 (135°). The order of the indices in the third level is *V*/8, 5*V*/8, 3*V*/8, and then 7*V*/8. The value of the index is rounded down to the nearest integer if the division results in a decimal. This process is repeated until all *L* levels are complete. This system was originally developed for a set of projections that covers the range 0 to 180°. For the projection set that covers a full rotation, the scheme would be used to calculate the order for the projections that cover the first 180°. The indices for the set of projections that cover the 180 to 360° range can be found by adding 180 to the set of indices covering the first 180°. No change needs to be made to (2) when using the MAS ordering scheme. To implement the MAS scheme, only the indices *A* and *B* need to be redefined so that *A* = (*v* − 1)*K* and *B* = *vK* where *v* is the projection view index determined according to the MAS scheme.

#### 2.2.3. Image Reconstruction for Dual-Source Dual-Detector TBCT

In the dual-source dual-detector configuration, four projection images are generated at each rotation angle. Each of the projection images collected at a given rotation angle is truncated both longitudinally and transversely as can be seen in the diagram shown in Figure 1(b). This leads to the center region of the FOV being covered by more than one source array detector array pair. Sequentially, backprojecting equally weighted correction terms calculated from each of the four projection images will cause artifacts. Instead, the correction terms from each of the four projection images are first weighted and then simultaneously backprojected onto the image. The weights applied to the correction terms for image voxel *i* are given by

(3) $$\begin{array}{c} w_{1,1,i}=\frac{e^{-k_{x^{\prime }}x_{i}^{\prime }}e^{-k_{z}z_{i}}}{\left(1+e^{-k_{x^{\prime }}x_{i}^{\prime }}\right)\left(1+e^{-k_{z}z_{i}}\right)}\\ w_{1,2,i}=\frac{e^{-k_{x^{\prime }}x_{i}^{\prime }}}{\left(1+e^{-k_{x^{\prime }}x_{i}^{\prime }}\right)\left(1+e^{-k_{z}z_{i}}\right)}\\ w_{2,1,i}=\frac{e^{-k_{z}z_{i}}}{\left(1+e^{-k_{x^{\prime }}x_{i}^{\prime }}\right)\left(1+e^{-k_{z}z_{i}}\right)}\\ w_{2,2,i}=\frac{1}{\left(1+e^{-k_{x^{\prime }}x_{i}^{\prime }}\right)\left(1+e^{-k_{z}z_{i}}\right)}, \end{array}$$

where *w*_*α*,*β*,*i*_ is the weight applied to the update term from the projection set collected using source array *α* and detector array *β*. *x*_*i*_′ is the transverse position of voxel *i* in the rotated reference frame, *z*_*i*_ is the longitudinal position of voxel *i*, *k*_*x*′_ is a constant used to vary the rate at which the transverse contribution fades out, and *k*_*z*_ is the constant used to vary the rate at which the longitudinal contribution fades out. Therefore, the new expression for the update term for the SART reconstruction method using the dual-source dual-detector configuration is

(4) xin+1=xin+λ   ×∑α=1 : 2∑β=1 : 2wα,β,i∑j=ABaij((pj−p^j)/∑i=1Naij)∑j=ABaij,

where *w*_*α*,*β*,*i*_ is the weighting factor defined by (3), *a*_*i*,*j*_ is an element of the system matrix **A**_*α*,*β*_, *p*_*j*_ is an element of the measured projection data set **p**_*α*,*β*_, and ${\hat{p}}_{j}$ is an element of the estimated projection set calculated using the system matrix **A**_*α*,*β*_.

### 2.3. Evaluation Method

#### 2.3.1. System Parameters

We employed the same geometry that was used in our TBCT benchtop system [13]. The reconstructed images had dimensions 256 × 256 × 128 with an isotropic voxel size of 1 mm. A total of 360 projections were generated at one degree intervals. For a TBCT system that incorporates a multirow detector array, each TBCT projection is a 3D matrix whose dimensions correspond to the number of sources, the number of detector columns, and the number of detector rows. Therefore, the TBCT projection data dimensions for our system containing 75 field emission X-ray sources and five detector rows with 275 detector columns per row were 75 × 275 × 5. The X-ray source spacing was 4 mm, and the isotropic detector pixel size was 2.54 mm. These projections were reconstructed using a modified FDK filtered backprojection algorithm and the SART algorithm both with and without the MAS ordering scheme.

#### 2.3.2. Phantom

The three-dimensional Shepp-Logan phantom [31] was used to test the performance of the reconstruction algorithms. The parameters were taken from this reference except that the density values of the ellipsoids were magnified to increase the contrast. Patient projection data were also generated by forward projecting the CT image of a real patient. The same matrix that was used for image reconstruction was also used to forward project the patient image for generation of the patient projection set. The resolution of the reconstructed image was 1 mm × 1 mm × 1.5 mm.

It has been shown that the use of the SART algorithm can mitigate the large cone angle artifacts that are produced when using approximate reconstruction methods such as the FDK algorithm [11]. In order to test the effectiveness of our modified algebraic reconstruction methods at reducing the cone angle artifacts, a numerical Defrise-like phantom was created [32]. The seven identical, longitudinally stacked ellipses of uniform density that compose this phantom provided a cone angle of 20 degrees. The phantom was positioned at the isocenter, which was set to be equidistant from both source and detector positions. The distance from the source to the detector was set at 64 cm. 

**Figure 2:**
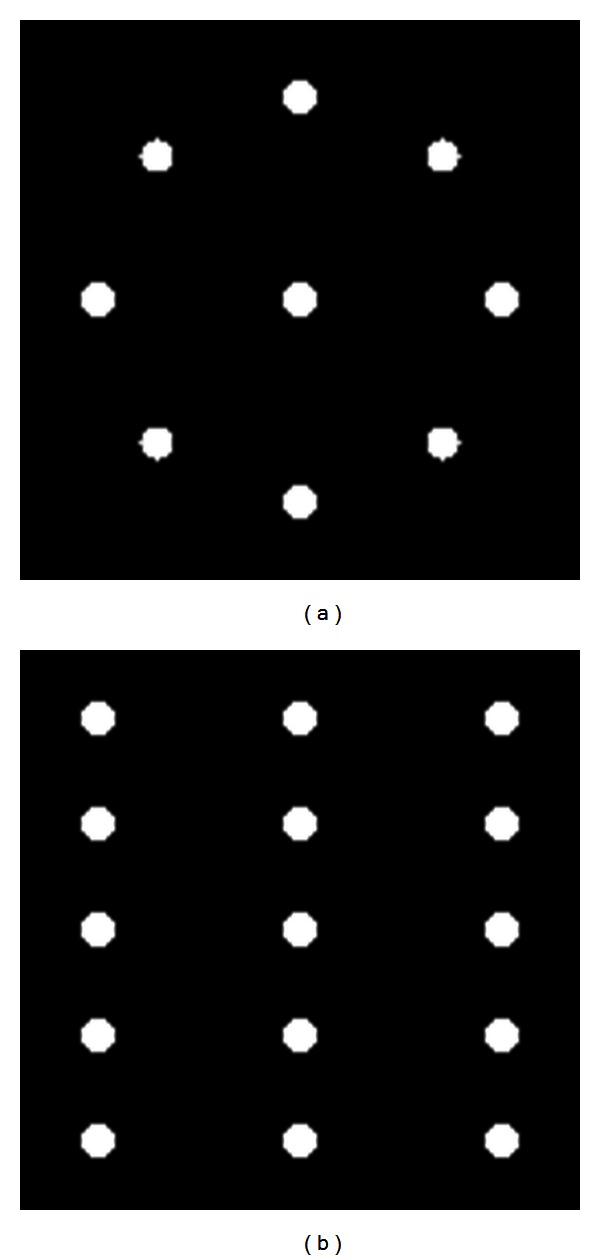
Transverse (a) and coronal (b) views of the spherical phantom.

**Figure 3:**
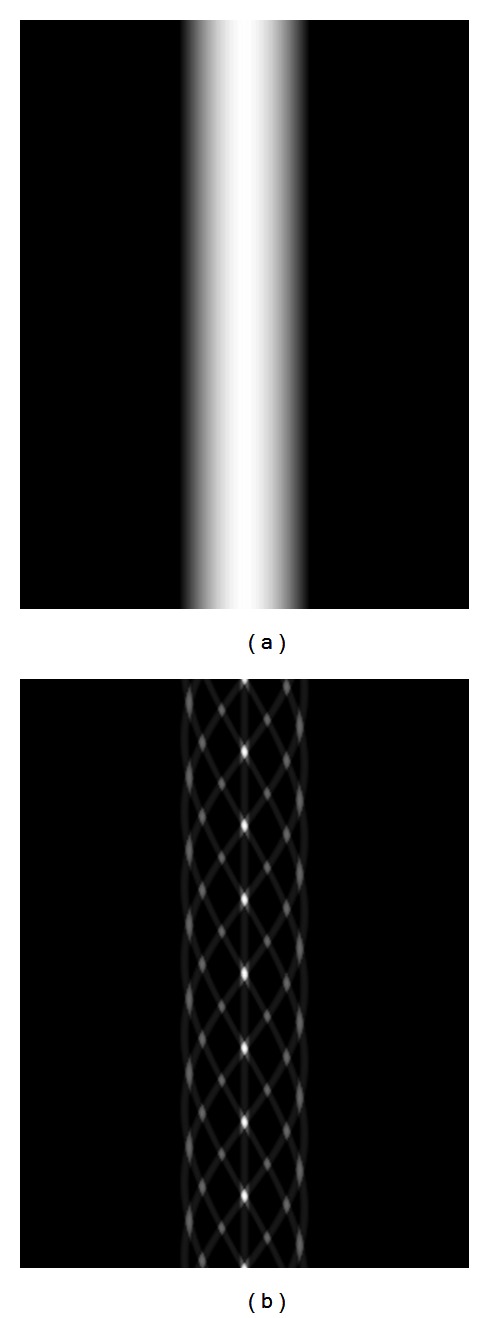
CBCT sinograms for the central slice of the (a) disk phantom and the (b) sphere phantom.

The linear system of equations when using the disk phantom has a very low rank due to the longitudinal symmetry of the phantom. We believe that the cone artifacts appearing in the reconstructions are exaggerated due to the atypical geometry of the disk phantom. In reality, the symmetry and shape of the disks do not appear in regular patients' images. To test the cone artifact using a phantom with a different configuration, we created a phantom where each disk was replaced by a set of nine small spheres. For this configuration, there is one central sphere and eight spheres equally spaced in a circle pattern around it as shown in Figure 2. Five sets of these sphere configurations were stacked longitudinally at equal intervals and together provided the same 20 degree cone angle that was provided by the disk phantom. Similar to the disk phantom, the sphere phantom is also longitudinally symmetric, but while the disk phantom generated identical projection images at every projection angle, the sphere phantom generated data that was sinusoidally varying as a function of projection angle.  Figure 3 compares the sinograms of the central slices for the two phantoms. The disk and sphere projection data were generated using the same method that was used to generate the Shepp-Logan projection data.

#### 2.3.3. Image Evaluation Metrics

The figures of merit (FOM) chosen for quantitative evaluation of the reconstructed images are the relative root mean square error (RRME) and the square Euclidean distance, which are defined by

(5) RRME=∑i∈I(xi−xiref)2∑i∈I(xiref)2,sqEuc=1−1N∑i∈I(xi−xiref)2,

where *x* ^ref^ is the reference image. 

## 3. Results

### 3.1. Evaluation of the Algebraic Reconstruction Methods

**Figure 4:**
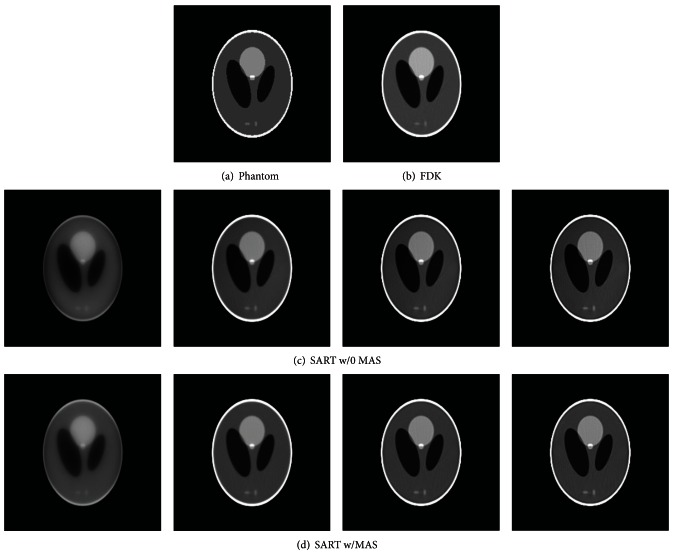
Reconstructions of the (a) Shepp-Logan phantom using the (b) FDK, (c) SART without the MAS ordering scheme, and (d) SART with the MAS ordering scheme. The four columns in (c) and (d) show reconstructed images after 1, 5, 10, and 15 iterations for the SART methods. The display window is set to [0.99 1.05].

**Figure 5:**
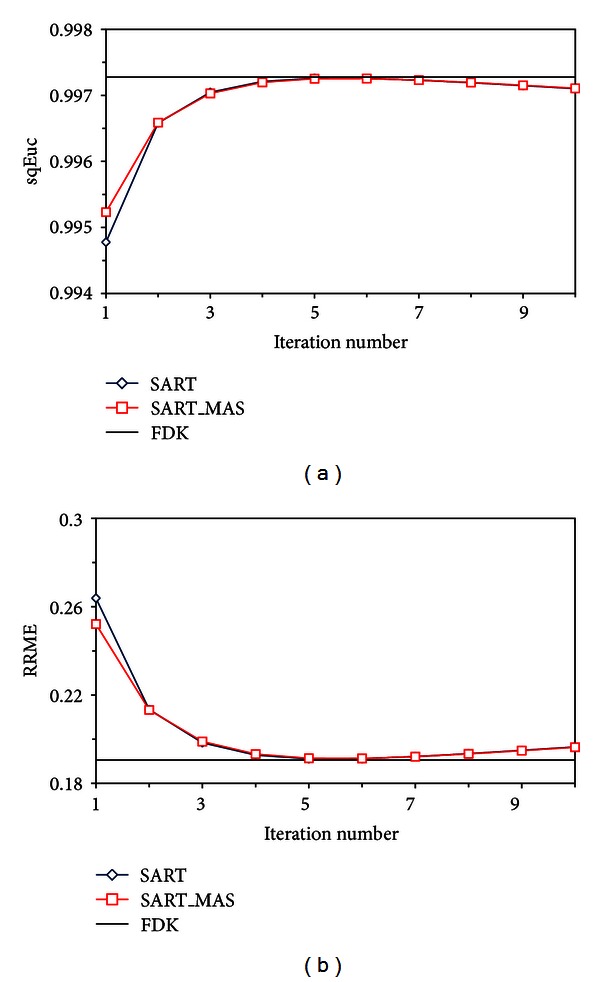
Comparison of convergence rates of three algebra reconstructions with (a) square Euclidean distance and (b) RRME.

We first tested the SART algorithms with data created using the [single-source single-detector] TBCT geometry. SART reconstructions of a transverse slice of the Shepp-Logan phantom after 1, 5, 10, and 15 iterations are displayed in Figure 4. The results are given for the SART method both with and without the MAS ordering scheme. The numerical phantom image and FDK reconstruction are also displayed for comparison. In Figure 5, the convergence rates of the algebraic methods are compared using the square Euclidean distance and RRME. The FOM values for the FDK reconstruction are displayed as reference lines on the graphs. Both metrics indicate that the algebraic methods achieved their best results between four and six iterations and that the FOM values for the algebraic methods were comparable to the FDK results in that region.

**Figure 6:**
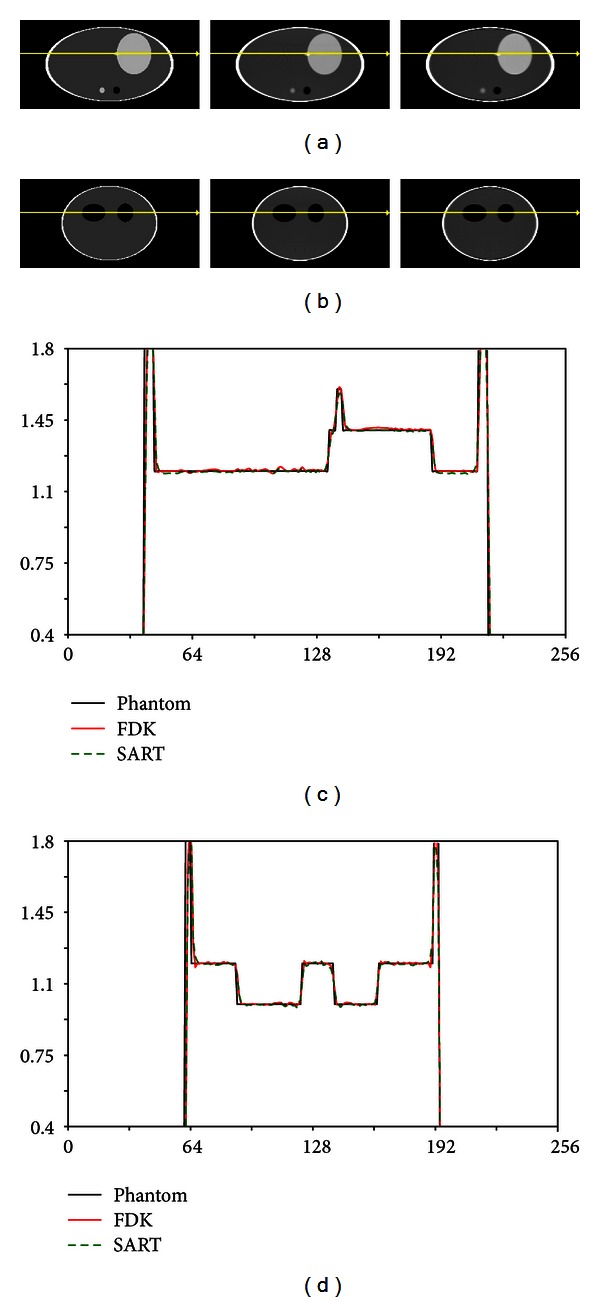
Reconstruction of noiseless Shepp-Logan data. (a) Sagittal and (b) coronal views of reconstructed images. (c) Sagittal and (d) coronal profiles of reconstructed images. In (a) and (b), from left to right are phantom, FDK reconstruction, and SART reconstruction images. For both the coronal and sagittal SART reconstructions, overshoot artifacts can be observed at the top and bottom edges of the phantom.

The SART method converged at approximately the same rate whether or not the MAS ordering scheme was used, but the SART method using the MAS scheme clearly gave better initial results. After five iterations, the SART method with the MAS ordering scheme was chosen as the method for providing the best balance between convergence speed and image quality. The reconstructions using the FDK and SART with MAS algorithms were then compared to the original phantom by evaluating a line integral taken through different views of the image. As shown in Figure 6, the line profile results from the sagittal and coronal views show good agreement between the reconstructed images and the numerical phantom.

In the images reconstructed using the SART algorithm, ringing artifacts can be seen at both the top and bottom of the phantom in both the coronal and sagittal views. This artifact is a result of using a cubic voxel discretization of the image space [24, 33–35]. Because of discretization of the continuous object, the modeled object edges are blurred and therefore the projection values calculated after forward projecting the image voxels will not match the measured values. Ringing artifacts will then result in areas with very high image gradients because the corrections to these voxels will be incorrectly weighted, and the resulting overshoot and undershoot in the voxels at the edges will then propagate to the neighboring voxels that are under a non-negativity constraint during further iterations. This effect is usually controlled by selecting a number of iterations that qualitatively provide the best tradeoff between edge sharpness and ringing artifact. The simplest way to mitigate this effect is to use a finer grid size [34], but this would lead to an increase in the computational expense of the algorithm. Other possible methods used to reduce these artifacts include the use of a spherically symmetric basis for the voxels instead of the cubic basis that is conventionally used [33, 36] and the use of a smoothing method during reconstruction [35]. 

**Figure 7:**
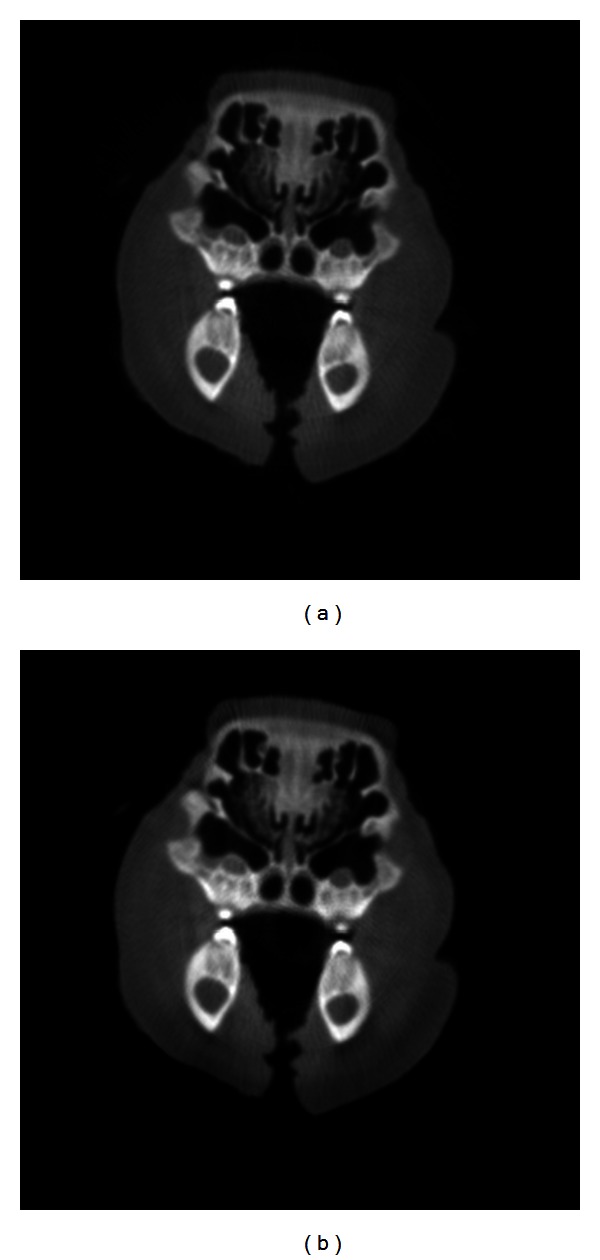
Transverse images of a pig's head with projections collected using the TBCT benchtop system and reconstructed using the (a) FDK algorithm and (b) SART algorithm with MAS scheme.

A pig's head was scanned using our TBCT benchtop system. The projections were reconstructed using the FDK and SART algorithms. With no image to use as a ground truth image, the reconstructions were evaluated qualitatively. Based on the results of the Shepp-Logan phantom, we used five iterations of the SART algorithm and used the MAS projection ordering scheme. A transverse image of the pig's head reconstructions is displayed in Figure 7 for comparison. There is close visual agreement between the images reconstructed using the different methods with slightly better contrast seen in the SART images.

### 3.2. Evaluation of the Cone Artifact

**Figure 8:**
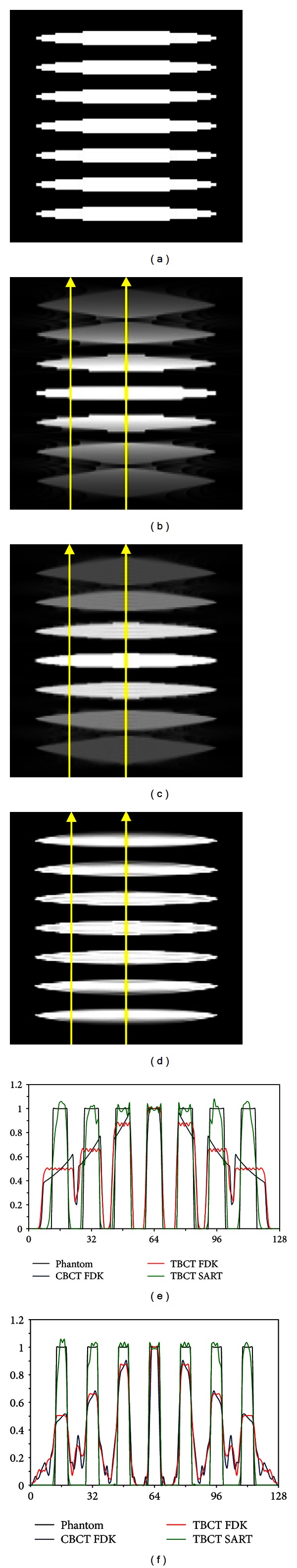
Comparison of reconstruction of a disk phantom with different methods. (a)–(d) Coronal views of original phantom, reconstructions using CBCT FDK, TBCT FDK, and TBCT SART methods, respectively. (e) and (f) are line profiles taken through the center and edges, respectively, of the images as indicated by the arrows.

Projection images of the Defrise-like phantom were generated for both CBCT and TBCT geometries. Coronal images of the original phantom as well as reconstructions produced using the regular FDK method for CBCT, the modified FDK method for the TBCT, and the SART with MAS method for TBCT are shown in Figures 8(a)–8(d). Line profiles taken through the center and edges of the coronal image are displayed in Figures 8(e) and 8(f), respectively. When using the FDK method, cone artifacts appeared in the reconstructions at the larger cone angles when using either of the TBCT and CBCT geometries. By contrast, the reconstructions produced using the SART algorithm did not suffer from large cone angle artifacts. There was, however, slight elongation of the disks along the longitudinal axis and a corresponding drop in CT values at the edges of the disk, though not to the extent experienced by the FDK reconstructions. 

**Figure 9:**
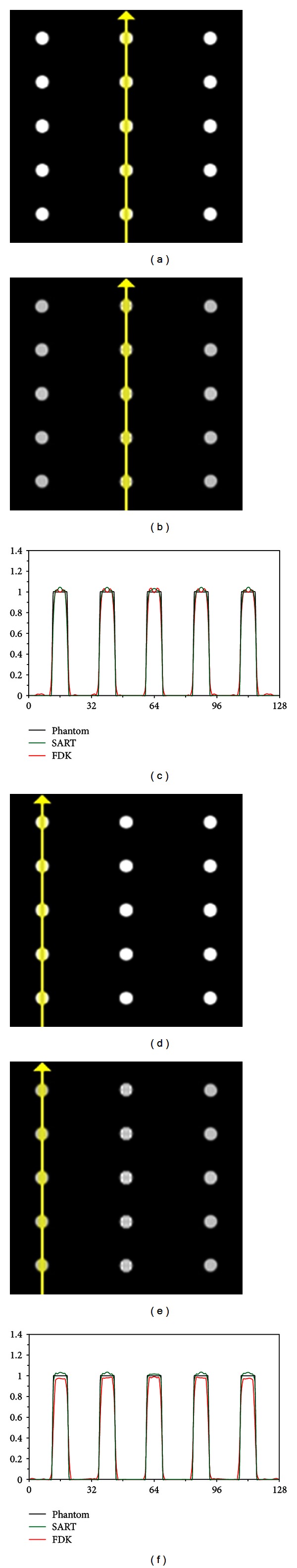
Coronal views of (a) SART and (b) FDK reconstructions with a (c) line profile taken through the center column. (d) SART and (e) FDK reconstructions with a (f) line profile taken through a side column.

The sphere phantom was reconstructed using the FDK and SART with MAS algorithms that were modified for use with the TBCT system. The TBCT system dimensions that were used for the disk phantoms were also used here.  Figure 9 shows the coronal views of the FDK and SART reconstructions. Line profiles were taken through the central column of spheres to verify that there is neither elongation nor decay in CT values for the spheres along the longitudinal axis. A slight elongation of the disks could still be observed in the FDK reconstruction but not in the reconstructed images produced by the SART algorithm. To check that the CT values were constant at the borders of the image, a line profile was also taken through a side column of spheres. The results were generally consistent with those obtained from the line profile through the central column, but the FDK reconstructions showed a slight drop in CT values toward the edges.

**Figure 10:**
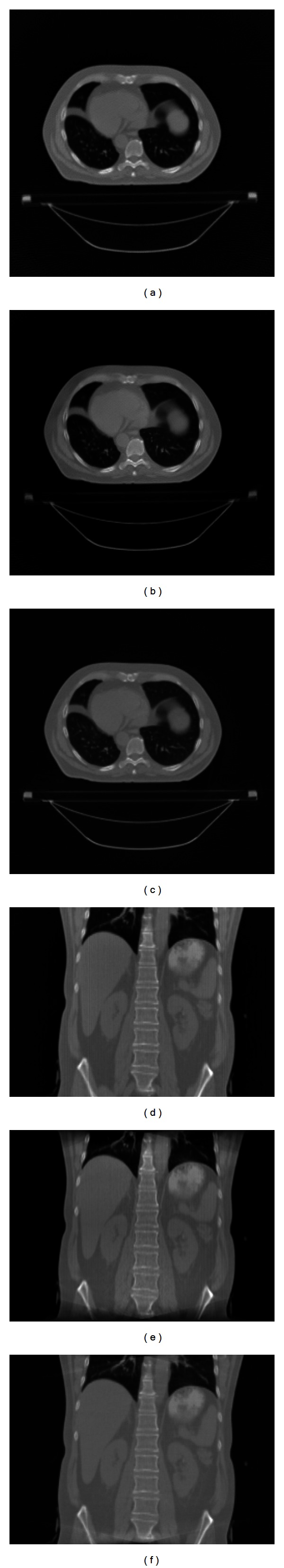
Reconstructed (top row) transverse images of a patient using the (a) fan-beam, (b) FDK, and (c) SART algorithms. Reconstructed (bottom row) coronal images using the (d) fan-beam, (e) FDK, and (f) SART algorithms.

We further tested the reconstruction methods using a patient image that was originally reconstructed using a diagnostic CT scanner. We generated TBCT projection data using the system geometry parameters of our benchtop TBCT system. Because the inherent resolution of the original patient image would be different than that of the TBCT reconstruction due to the differences in the scanning geometry used to create our projections as opposed to the scanning geometry used with the diagnostic CT scanner, we generated a projection set using a fan-beam geometry that had exactly the same system dimensions as the central plane of our TBCT system. The resulting fan-beam reconstruction had the same inherent resolution as our TBCT system and could therefore be used for comparison with the TBCT reconstructions. The projections were reconstructed using the fan-beam filtered backprojection (FBP) algorithm, the FDK algorithm modified for the TBCT geometry, and the SART with MAS algorithm for TBCT. The reconstructed images had dimensions 256 × 256 × 123 with voxel dimensions of  2 mm × 2 mm × 3 mm. Figures 10(a) and 10(d) show the transverse and coronal images, respectively, of the FBP algorithms using the simulated helical CT data. The cone angle to the outermost slices was 25°. Figures 10(b) and 10(e) show the transverse and coronal images, respectively, reconstructed using the modified FDK algorithm on the simulated TBCT data, and Figures 10(c) and 10(f) show the same two images after five iterations of the SART algorithm also using the simulated TBCT data. No elongation is apparent in the images produced using either the FDK or SART algorithms. The coronal images demonstrate that the TBCT reconstructions do not show any noticeable elongation for objects at higher cone angles during a patient scan. These results are consistent with the testing performed on the sphere phantom. 

### 3.3. Iterative Reconstruction for Dual Source-Dual Detector TBCT

**Figure 11:**
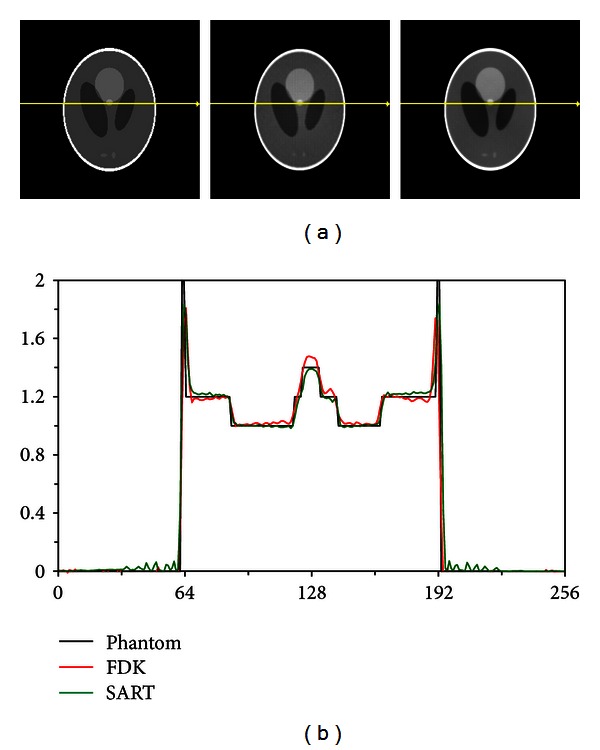
Reconstructions (a) of the (left) Shepp-Logan phantom using the (center) FDK filtered backprojection algorithm and the (right) SART algorithm with (b) a line profile taken through the reconstructions.

The dual source-dual detector TBCT configuration is preferable for image-guided radiotherapy. However, for this configuration, the maximum cone angles are in the region of the central axis, and therefore, the large angle artifacts will be most significant at the center of the reconstructed image. Moreover, the detector and source arrays do not cover the full FOV so that the data is truncated both longitudinally and transversely. We used the Shepp-Logan phantom and clinical CT images to test the performance of the TBCT and SART algorithms that were modified for the dual source-dual detector TBCT geometry. The detector array was modified so that the projection data associated with each source array detector array pair had dimensions 75 × 200 × 5. The size of the detectors was kept the same, and the reconstructed image dimensions were 256 × 256 × 128 with an isotropic voxel length of 1 mm. We performed five iterations of the SART algorithm while using the MAS scheme and compared the results with those of the modified FDK algorithm. As seen in Figure 11, the image was reconstructed without the addition of significant artifacts that would have been caused by either longitudinal or transverse truncation.

**Figure 12:**
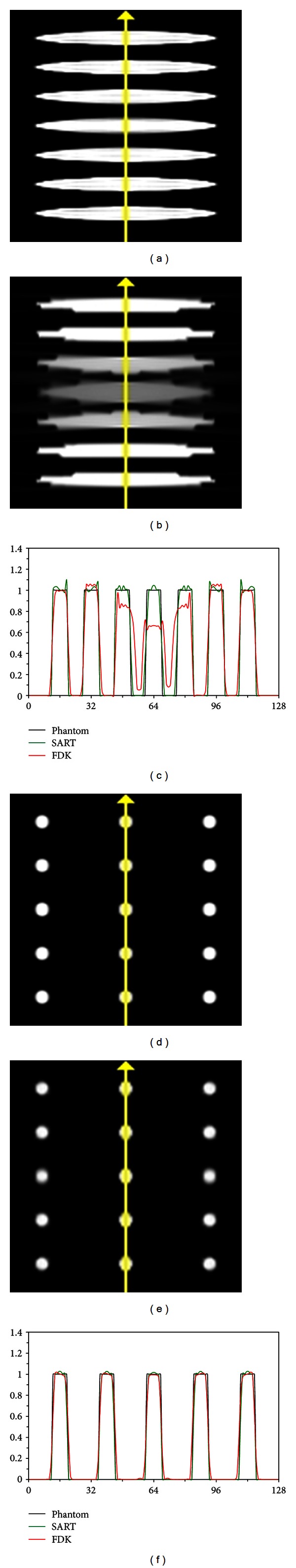
Transverse images reconstructed using the (a) FBP algorithm on data generated using fan-beam geometry and the (b) SART algorithm using the dual-source dual-detector geometry. Coronal images were also reconstructed using the (c) FBP and (d) SART algorithms.

To further evaluate the performance of the algorithms at large cone angles, we used the same disk and sphere phantoms defined above. The system parameters that were used to generate the data sets were kept constant. The reconstruction results are shown in Figure 12. As expected, the cone angle artifact in the FDK reconstruction increased towards the center of the image where the angle was the largest, while no artifact was observed in the SART reconstruction. There was a slight elongation of the disks that appeared around the central slice for both algorithms, but it was more pronounced in the FDK reconstruction. The reconstructions of the sphere phantom showed neither elongation nor cone artifacts when using the SART algorithm, but there was a slight elongation around all the spheres in the reconstructions produced by the FDK algorithm.

**Figure 13:**
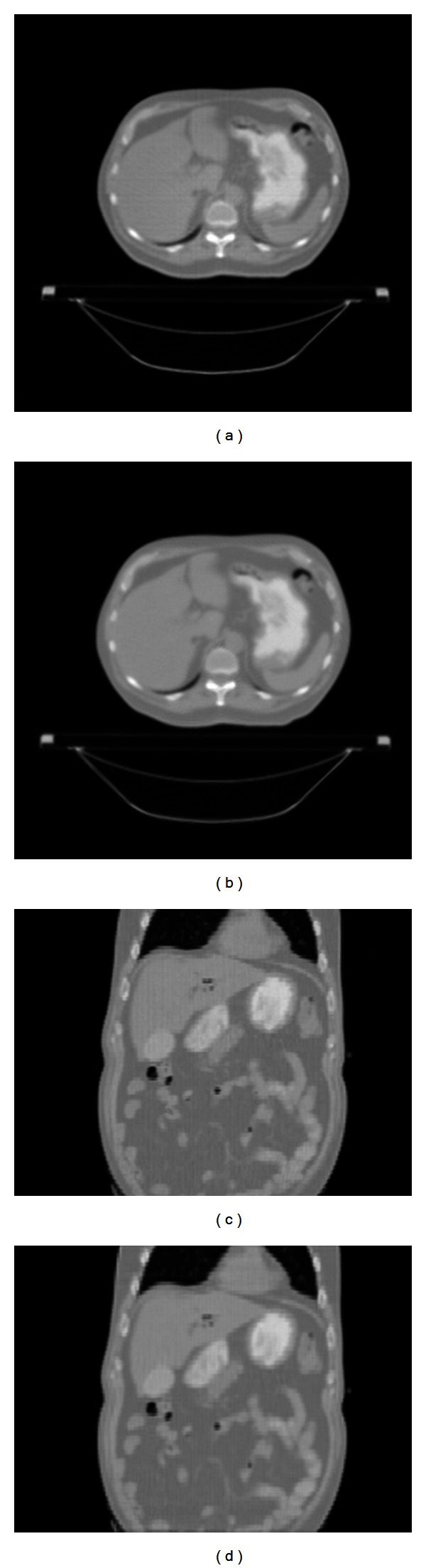
Transverse patient images reconstructed using the (a) FBP algorithm on data generated from fan beam geometry and the (b) SART algorithm from data generated using the dual source-dual detector geometry. Coronal images were also reconstructed using the (c) FBP and (d) SART algorithms.

Similarly, a diagnostic CT image was then used to create the four simulated projection sets that would be produced by a dual source-dual detector TBCT system. As shown in Figure 13, there are no significant artifacts introduced into the image due to the transverse truncation. Because the inherent resolution of the patient image would again have been different due to the use of different scanning parameters, we again used the same system parameters that were used for the central plane of our benchtop system to generate a fan-beam projection set. The spatial resolution of the image reconstructed using this new fan-beam projection set was comparable to that provided by the SART reconstruction. 

## 4. Discussion and Conclusion 

In this paper, the FDK and SART methods were implemented for the TBCT geometry. Data generated using numerical phantoms and clinical CT images as well as data collected using our TBCT benchtop system were reconstructed with these modified methods. The accuracy of the FDK and SART reconstructions was evaluated using the square Euclidean distance and the root mean square error FOMs. For small cone angles, the algebraic SART methods for both TBCT geometries were able to provide image quality comparable to that of the analytical FDK algorithm. For large cone angles, use of the algebraic image reconstruction algorithms significantly reduced the cone artifacts that were especially prominent in the FDK phantom reconstructions. This was especially important for the dual source-dual detector TBCT geometry as the cone artifacts were most prominent in the center of the image for this geometry. The results given by implementing the SART method are promising, and the algorithm may be implemented in future TBCT systems. 

The use of model-based statistical iterative image reconstruction methods, which can more accurately model the physics of the system and better take into account sources of noise and the statistical distribution of that noise, can potentially further improve the image quality. Because of their accurate modeling of the system geometry and data collection process, we expect that model-based statistical iterative image reconstruction methods can mitigate cone artifacts in a similar way as the SART method. Therefore, a model-based statistical reconstruction method that incorporates an accurate model of the system geometry and physics into the calculation of the system matrix is planned for a future study.

The use of the MAS ordering scheme improved the accuracy of the reconstruction during the first few iterations of the SART method, particularly in the first iteration. After that, the reconstruction results both with and without using the MAS scheme were almost indistinguishable. One reason that the MAS scheme did not increase the convergence rate or accuracy after the initial iterations may be that the scheme was originally designed for a parallel-beam configuration. The most straightforward method that could be attempted to improve the performance of the MAS scheme would then be to rebin the fan-beam data in order to create a parallel beam projection data set. However, rebinning the data may unnecessarily complicate the calculation of the system matrix when we transition to model-based iterative reconstruction. Therefore, we kept the fan-beam geometry for this study. 

The speed of the reconstruction method must improve for future implementation of the algorithm. The large computational burden required when employing iterative reconstruction algorithms is the main reason that it has taken so long for these methods to be implemented in the clinic. Using this algorithm, reconstructions would take as long as two hours to complete. However, the literature shows promising results from the implementation of iterative algorithms using graphics processing units (GPU). Acceleration in reconstruction times on the scale of one to two orders of magnitude have been reported [20, 28, 37, 38]. For these methods, though, the system matrix is much too large to hold in the GPU's memory and, therefore, must be calculated during runtime. The use of a spherical pixellation scheme has been used to take advantage of the symmetries of the circular scanning geometry of the system in order to reduce the storage requirement of the system matrix and to, therefore, improve the speed of reconstruction [39]. Therefore, the development of a cylindrical voxelization scheme in a separate study could potentially accelerate the reconstruction process on its own or, by reducing the size of the system matrix, make it feasible to implement the method on the GPU. 

In conclusion, algebraic iterative reconstruction algorithms were successfully implemented for the TBCT system. The analytical and iterative reconstructions showed similar image quality for reconstructions at small cone angles, while the iterative methods were able to mitigate the cone artifacts that normally appear at large cone angles in analytical methods. The iterative algorithms were also able to accurately account for both longitudinal and transverse truncation of the projection data without introducing new artifacts into the image.
